# Large buoyant particles dominated by cyanobacterial colonies harbor distinct bacterial communities from small suspended particles and free‐living bacteria in the water column

**DOI:** 10.1002/mbo3.608

**Published:** 2018-03-23

**Authors:** Limei Shi, Yaxin Huang, Min Zhang, Xiaoli Shi, Yuanfeng Cai, Shengling Gao, Xiangming Tang, Feizhou Chen, Yaping Lu, Fanxiang Kong

**Affiliations:** ^1^ State Key Laboratory of Lake Science and Environment Nanjing Institute of Geography and Limnology Chinese Academy of Sciences Nanjing China; ^2^ Biological Experiment Teaching Center College of Life Sciences Nanjing Agricultural University Nanjing China; ^3^ State Key Laboratory of Soil and Sustainable Agriculture Institute of Soil Science Chinese Academy of Sciences Nanjing Jiangsu Province China

**Keywords:** *Actinobacteria*, cyanobacterial aggregates, illumina sequencing, particles

## Abstract

Worldwide cyanobacterial blooms greatly impair ecosystems in many eutrophic lakes and impact the microbial environment. In particular, large cyanobacterial colonies that are buoyant on the water surface may provide a distinct habitat for bacteria from other small particles that are suspended stably in the water column. To test this hypothesis, bacterial communities (excluding cyanobacteria) attached to large particles dominated by cyanobacterial colonies (>120 μm, LA), small particles (3–36 μm, SA), and free‐living bacteria (0.2–3 μm, FL) were investigated monthly for a year in Lake Taihu, China. Results confirmed that the Shannon diversity index of LA was significantly lower than that of FL, which was lower than that of SA. *Cytophagia* and *Alphaproteobacteria* were specially enriched in LA. Although samples in each habitat collected during high‐ (May to November) and low‐bloom seasons (December to April) were separated, all samples in LA were clustered and separated from SA and FL, which were also clustered during the same sampling seasons. In addition, the bacterial communities in LA were correlated with nitrate level, whereas FL and SA were correlated with nitrate level and temperature. Mantel analysis revealed that bacterial composition significantly correlated with the cyanobacterial composition in LA and FL but not in SA. These results indicate that LA provides distinct niches to bacteria, whereas the differentiation of bacterial communities in FL and SA is seasonally dependent.

## 1 INTRODUCTION

Cyanobacterial blooms dominated by *Microcystis* and *Dolichospermum* (*Anabaena*) widely occur in eutrophic lakes worldwide (Paerl & Otten, 2013). Such blooms undergo recruitment, formation, maintenance, and decline from the spring to winter seasons in temperate lakes (Kong & Gao, 2005). However, along with global change and human activities, the duration of cyanobacterial blooms has prolonged from only covering warm seasons previously to currently extending to almost an entire year. Such drastic alteration has greatly changed the entire ecosystem in Lake Taihu, a typical eutrophic lake in China (Qin, Xu, Wu, Luo, & Zhang, 2007). The long‐term duration and high biomass of cyanobacterial blooms result in the domination of cyanobacterial colonies in the lake's particle composition (Shi et al., 2017). Compared with other abiotic particles and the water column, these colonies are rich in organic matter produced by cyanobacterial cells and offer a “hotspot” for bacterial colonization (Worm & Sondergaard, 1998). Interactions such as nutrient transference (Yuan, Zhu, Xiao, & Yang, 2008), growth promotion or inhibition (Berg et al., 2009; Shi, Cai, Li, et al., 2009; Xie et al., 2016), and toxin production and degradation between bacteria and cyanobacteria are intense in these colonies (Maruyama et al., 2003). Furthermore, these bacteria are closely associated with cyanobacterial bloom formation and nutrient cycling, which greatly affect the development and decline of cyanobacterial blooms (Rashidan & Bird, 2001; Wang, Zhang, Shen, Xie, & Yu, 2015; Wang et al., 2016).

In particular, large cyanobacterial colonies exhibit different physiological and ecological characteristics from small particles composed of eukaryote algae, single‐cell/small cyanobacterial colonies, algal detritus, and abiotic particles. First, cyanobacterial colonies larger than 120 μm are often buoyant on water surface (Wu et al., 2010), whereas small particles encounter more difficulty rising onto the water surface and are mainly suspended in the water column (Wu & Kong, 2009; Zhu et al., 2014). Second, large *Microcystis* colonies (>100 μm) have higher proportions of microcystin‐producing genotypes, whereas the smallest size class of *Microcystis* colonies (<50 μm) has a low proportion of microcystin‐producing genotypes (Kurmayer, Christiansen, & Chorus, 2003; Wang et al., 2013). This case is similar to that for other small particles. Third, compared with small particles suspended in the water column, large cyanobacterial colonies have higher polysaccharide content, are more effectively resistant to high‐light inhibition (Zhang, Shi, Yu, & Kong, 2011), have higher affinity for low levels of phosphorus (Shen & Song, 2007), and have stronger defense against grazing (Nielsen, 2006; Yang, Kong, Shi, & Cao, 2006). Small particle‐attached bacteria may face a considerably different environment from that embedded in large buoyant cyanobacterial colonies. Furthermore, bacteria free living in the water column may be affected by dissolved substances, such as dissolved organic matter and toxins. Therefore, the compositions of bacterial community attached to large cyanobacterial colonies and small suspended particles may differ due to different characteristics, compositions, and positions in the water column; they may also be different from free‐living bacteria in the water column (Schmidt, White, & Denef, 2016).

Many works have focused on bacterial communities associated with cyanobacterial blooms (Berg et al., 2009; Berry et al., 2017; Dziallas & Grossart, 2011; Eiler & Bertilsson, 2004; Niu et al., 2011; Tang et al., 2010; Woodhouse et al., 2016). Some works revealed the transition of particle‐attached bacteria to free‐living bacteria during a 4‐month investigation (Tang et al., 2015, 2017), and others investigated the difference between particle‐attached bacteria and free‐living bacteria (Zhao et al., 2017). The bacteria attached to medium‐ and small‐sized cyanobacterial aggregates during August and September were clustered, whereas large‐ and medium‐sized aggregate communities in the October sample were grouped together and appeared distinct from the small‐sized aggregate community (Cai, Jiang, Krumholz, & Yang, 2014; Cai, Yan, Wang, Krumholz, & Jiang, 2013). Different bacteria attached to *Microcystis* colonies from free‐living bacteria for several months were also documented (Parveen et al., 2013; Shi, Cai, Kong, & Yu, 2012). However, bacteria involved in three habitats, including large buoyant cyanobacterial colonies, small particles, and free‐living bacteria in the water column, have yet to be compared in a duration of 1 year. Therefore, whether this controversial conclusion results from the lack of differentiation among these habitats or sampling season still needs to be determined. In addition, the detailed response of bacterial communities from different habitats to cyanobacterial blooms remains unknown.

In this study, we resolved this problem in Lake Taihu, which is shallow eutrophic, well‐mixed, and known for having a long history of cyanobacterial blooms (Qin et al., 2007). Satellite images revealed that cyanobacterial blooms are even more intense and occur year‐round in Lake Taihu, especially in Meiliang Bay, which is a hypertrophic area of the lake (Ma et al., 2016). Thus, we can obtain large cyanobacterial colonies even in winter. Our goal is to determine whether large buoyant cyanobacterial colonies and small particles provide different habitats for bacterial communities and how they are different from free‐living bacteria.

## 2 MATERIALS AND METHODS

### 2.1 Sample site description

Lake Taihu is located in the Yangtze Delta in Eastern China (30°55′40″–31°32′58″N, 119°52′32″–120°36′10″E). With a total area of 2338 km^2^ and an average depth of 2 m, Lake Taihu is the third largest freshwater lake in China. With increased nutrient inputs into the lake during the past decades, Lake Taihu is eutrophic with blooms of *Microcystis* (cyanobacteria) occurring annually during warm seasons (Chen, Qin, Teubner, & Dokulil, 2003). Meiliang Bay, located in the northern part of Lake Taihu (Figure S1), is the most eutrophic area where cyanobacterial blooms break out and last for almost a whole year (Ma et al., 2016; Qin et al., 2007).

### 2.2 Sampling procedure

Sampling was performed monthly from July 2014 to July 2015 near the regular monitoring stations of the Taihu Laboratory for Lake Ecosystem Research in Meiliang Bay of Lake Taihu. Because Taihu Lake is shallow and well‐mixed (McCarthy et al., 2007), sampling of the following two parts was considered as originated from the same environment. For bacteria attached to large cyanobacterial colonies (LA, >120 μm), samples were collected by towing a phytoplankton net (64 μm mesh) through water surface, then the top buoyant cyanobacterial colonies were pipetted and filtered through 120 μm mesh net, and then collected into sterile polypropylene tubes. For bacteria attached to small particles (SA, 3–36 μm) and free‐living bacteria (FL, 0.2–3 μm), lake water at 0–0.5 m depth was collected with a water sampler, and was sequentially filtered through 36 μm mesh, 3 and 0.2 μm polycarbonate filters (GTTP, 47 mm diameter; Millipore). The tubes and filters were immediately frozen in liquid nitrogen. Water temperature, pH, and dissolved oxygen (DO) were monitored with a multiparameter meter (model 6600; Yellow Spring Instruments, OH, USA). For analysis of nutrient concentrations including ammonium (NH_4_^+^), nitrate (NO_3_^−^), nitrite (NO_2_^−^), and phosphate (PO_4_^3−^), water samples were filtered through precombusted GF/F glassfilbers (47 mm diameter; Whatman), and then the filtrates were analyzed with a continuous flow analyzer (Skalar San++, Netherlands). Total phosphorus (TP) and total nitrogen (TN) were quantified using standard methods (Jin & Tu, 1990). Chlorophyll *a* (Chl *a*) was extracted from the GF/F filters with 90% acetone and analyzed with a spectrofluorophotometer (RF‐5301PC; Shimadzu, Japan).

### 2.3 Microscopic examination of LA and SA

Particle composition of LA and SA, representative samples were examined under a JSM‐5610LV/Vantage IV scanning electronic microscope (SEM). All samples for SEM were prepared with direct freeze–drying to minimize changes on the cell surfaces induced by chemical reactions such as the chemical fixation and ethanol dehydration, and minimize the in‐hand manipulation time (Lee & Chow, 2012). The dried samples were then coated with gold and viewed on the SEM.

### 2.4 DNA extraction and MiSeq sequencing of the 16S rRNA genes

Nucleic acid extraction was conducted following the xanthogenate‐SDS extraction protocol (Tillett & Neilan, 2000). PCR amplification of the V3–V4 region of the 16S rRNA gene was performed with the bacterial universal primers 338F (5′‐barcode‐ACTCCTACGGGAGGCAGCAG‐3′) and 806R (5′‐GGACTACHVGGGTWTCTAAT‐3′) (Gohl et al., 2016; Lee, Barbier, Bottos, McDonald, & Cary, 2012). The barcode was an eight‐base sequence that is unique to each sample. PCR amplification was performed by GeneAmp^®^ PCR System 9700 (Applied Biosystems, USA). Thermal cycling conditions were as follows: initial denaturation at 95°C for 3 min, and 25 cycles at 95°C for 30 s, 55°C for 30 s, and 72°C for 45 s, with a final extension at 72°C for 10 min. Successful amplification was confirmed via agarose gel (1%) electrophoresis with 2 μL of PCR product. Purified amplicons were sequenced with Illumina Miseq PE300 by Majorbio Bio‐Pharm Technology Co., Ltd. (Shanghai, China). The sequence data were deposited in the National Center for Biotechnology Information (NCBI) Sequence Read Archive (http://trace.ncbi.nlm.nih.gov/Traces/sra/) under accession numbers SRP108467, BioProject PRJNA386411, and BioSamples SAMN07178582‐ SAMN07178620.

### 2.5 Statistical analyses

The raw data were first quality filtered with QIIME (Caporaso et al., 2010) to remove reads that did not meet the quality control standards. Any chimeric sequence was identified and removed with UCHIME (Edgar, Haas, Clemente, Quince, & Knight, 2011). Operational taxonomic units (OTUs) were clustered with 97% similarity cutoff using UPARSE version 7.1 http://drive5.com/uparse/) (Edgar, 2013). The taxonomy of each 16S rRNA sequence was analyzed by RDP Classifier (http://rdp.cme.msu.edu/) against the SILVA 16S rRNA database (SSU123; Max Planck Institute, Germany) using a 70% confidence threshold. Coverage, Chao 1 index (a species richness index), and Shannon index (a diversity index that accounts for abundance and evenness) were calculated with QIIME.

To compare the samples, the dataset was randomly subsampled to an equal number of sequences. Nonmetric multidimensional scaling (NMDS) analysis based on Bray–Curtis algorithm distance matrix was performed for all samples on the OTU level. At the same time, weighted pair group method for the arithmetic means (WPGMA) cluster analysis based on Jaccard's Coefficient analyzed using the MultiVariate Statistical Package (MVSP) software (Package 3.1; Kovach Computing Services, UK). Analysis of similarities (ANOSIM) was used to directly compare bacterial communities in three different habitats including LA, SA, and FL. Taxa that were significantly different between the three different habitats were detected using the Bioconductor‐edgeR package (version 3.2.4) (Robinson, McCarthy, & Smyth, 2010; Robinson & Oshlack, 2010). Distance‐based redundancy analysis (dbRDA) was used to examine the influence of detected environmental factors including NH_4_^+^, NO_3_^−^, NO_2_^−^, PO_4_^3−^, TOC, Chl *a*, temperature, pH, DO, and TN on the dynamics of bacterial communities. Significance of variables was assessed with Monte–Carlo permutation tests (999 unrestricted permutations). Mantel analysis with Bray–Curtis dissimilarity matrix was used to analyze correlation between cyanobacterial and bacterial composition in each habitat. All these analyses were performed with the “vegan” package (Oksanen et al., 2008) of R software (R Development Core Team 2012)A. Furthermore, to identify characteristic community members on genus level in the three different habitats (LA, SA, FL), we applied the linear discriminant analysis (LDA) coupled with effect size measurements (LEfSe) method (Segata et al., 2011).

## 3 RESULTS

### 3.1 Temporal dynamics of environmental factors and particle composition

**Figure 1:**
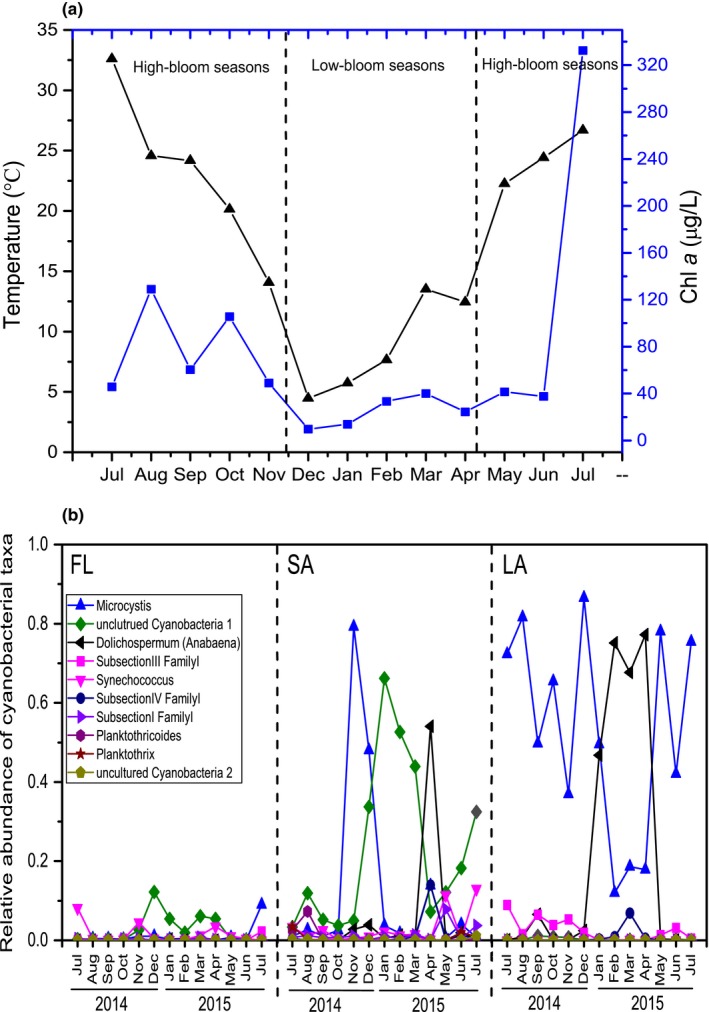
Dynamics of temperature, Chl *a*, and cyanobacterial composition. (a) Changes in temperature and Chl *a* concentration in the Meiliang Bay of Lake Taihu, China. (b) Relative abundances of cyanobacterial taxa in the three habitats during July 2014 and July 2015 in Meiliang Bay of Lake Taihu, China. LA represents large cyanobacterial colonies (>120 μm), SA represents small particles (3–36 μm), and FL represents free‐living bacteria (0.2–3 μm) in the water column

From July 2014 to July 2015, water temperature decreased gradually from 32°C in July to 5°C in December, and then gradually increased to 26°C (Figure 1a). Pearson correlation analyses revealed that pH was positively correlated with temperature (*R* = .864, *p *<* *.001), whereas DO was negatively correlated with temperature (*R* = −.686, *p *=* *.01). Chl *a* varied from 9.7 μg/L in December 2014 to 332.6 μg/L in July 2015 (Figure 1a), and was significantly correlated with TN (*R* = .735, *p *=* *.004) and TP (*R* = .788, *p *=* *.001). So, it was low‐bloom seasons from December to April, and was high‐bloom seasons from May to November in this lake.

**Figure 2:**
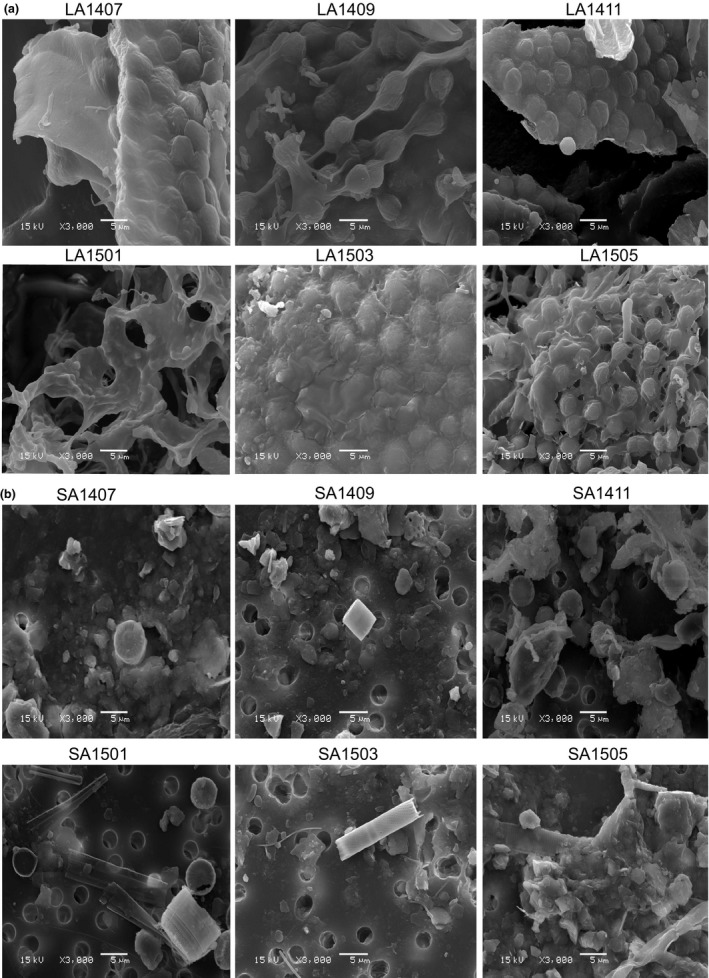
SEM examinations of LA (a) and SA (b). LA represents large buoyant cyanobacterial colonies (>120 μm), and SA represents small particles (3–36 μm). 1407, 1409, 1411, 1501, 1503, and 1505 are the representative samples collected in July, September, November 2014, and January, March, May 2015, respectively

SEM analysis confirmed that LA was overwhelmingly dominated by cyanobacterial colonies, whereas SA was dominated by diatoms, single‐cell/small cyanobacterial colonies, chlorophyta, algal detritus, and sediment particles. Bacteria attached to these particles can also be viewed via SEM (Figure 2).

### 3.2 Sequence analyses and cyanobacterial composition

We analyzed the sequence data at two levels and normalized the analysis before and after elimination of the cyanobacterial sequences. The primary result was normalized to 25,123 sequences in each sample. In LA, the relative abundances of cyanobacterial sequences in the total sequences varied from 43.2% to 96.7%, and the proportion tended to be higher from December to April (low‐bloom seasons) compared with other months (Figure 1b). In SA, the relative abundances of cyanobacterial sequences in the total sequences varied from 7.4% to 89.6%, which is a wider range than that in LA (Figure 1b). The proportion also tended to be higher from December to April (low‐bloom seasons) compared with other months; this result is similar to that in LA. Only 0.1%–13.8% of the total sequences corresponded to cyanobacteria in FL (Figure 1b). Furthermore, a shift of the dominant cyanobacterial taxa was observed. In LA, *Dolichospermum* was dominant from January to April (67.7%–77.2%) (low‐bloom seasons), whereas *Microcystis* was absolutely dominant during the other months (37.0%–86.5%) (high‐bloom seasons). However, in SA, *Dolichospermum* was only dominant in April (54.1%); *Microcystis* was dominant in November (79.3%) and December (48%); and uncultured cyanobacteria 1 was dominant during the other months (3.3%–66.2%). In FL, Subsection III Family I was dominant in October (0.3%–9%), *Microcystis* was dominant from August to September 2014 and July 2015, uncultured cyanobacteria 1 was dominant from December to April (1.9%–12.2%) (low‐bloom seasons), and *Synechococcus* was dominant during the other months (1.0%–8.2%) (Figure 1b).

### 3.3 Diversity of bacterial communities in three habitats (LA, SA, and FL)

**Figure 3:**
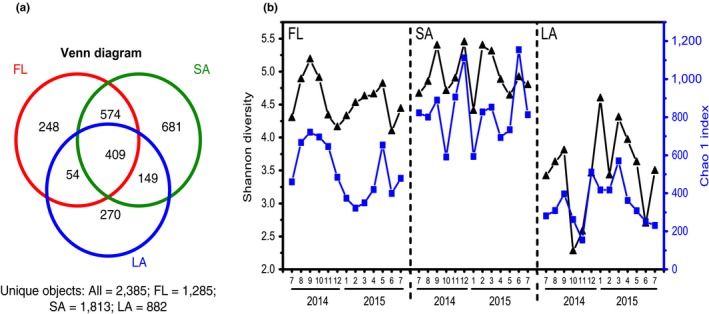
Bacterial phylogenetic alpha diversity. (a) Venn diagram showing the number and abundance of the bacterial OTUs found in the three habitats. (b) Shannon's diversity index and Chao 1 index calculated individually for each sample presented as line charts. LA represents large cyanobacterial colonies (>120 μm), SA represents small particles (3–36 μm), and FL represents free‐living bacteria (0.2–3 μm) in the water column

To compare the bacterial communities among the three habitats (LA, SA, and FL), cyanobacterial sequences were eliminated, and the remaining were normalized to 2,561 sequences for each sample. The estimated coverage values of all samples were still higher than 0.88 [data not shown, calculated by the equation *C *= (1 − *n*_i_/*N*), where *n*_i_ is the number of OTUs represented by one sequence and *N* is the number of sequences in each sample (Good, 1953)], suggesting a sufficient number of sequences for analyses that nearly embraced the entire dominant biodiversity. Among the 2385 OTUs for all samples, 17.1% (*n *=* *409) was shared among the three groups, whereas some OTUs occurred exclusively in either FL (*n *=* *248, 10.4%), LA (*n *=* *270, 11.3%), or SA (*n *=* *681, 28.6%). A total of 24.1% (*n *=* *574) were only shared between FL and SA, whereas only 2.2% (*n *=* *54) was only shared between LA and FL or only between LA and SA (*n *=* *149, 6.2%; Figure 3a). Shannon diversity and Chao1 indices were significantly higher in the bacterial communities of SA than in those of FL (using nonparametric Mann–Whitney *U* test, *p *<* *.001), which was higher than that of LA (using nonparametric Mann–Whitney *U* test, *p *=* *.003) (Figure 3b).

**Figure 4:**
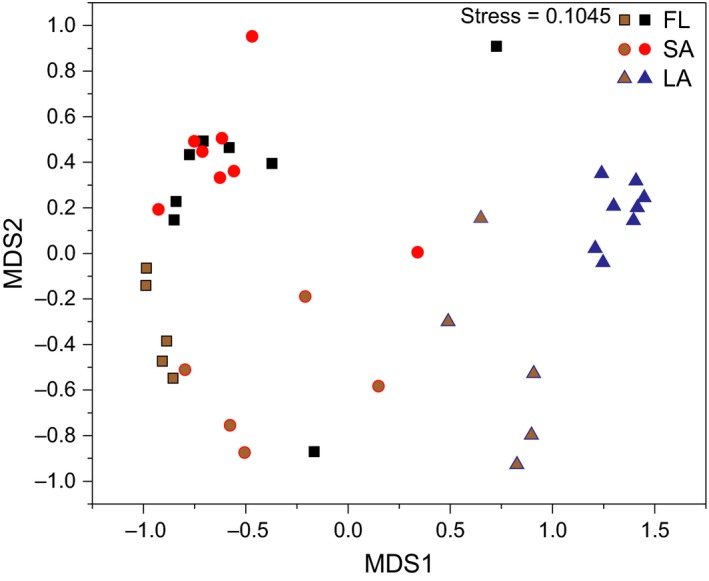
Non‐metric multidimensional scaling (NMDS) plot based on Bray–Curtis dissimilarity. The filled triangles, circles, and squares reflect bacterial community composition in the different samples corresponding to the different habitats LA, SA, and FL according to the legend. Symbols in brown color were the samples collected from December 2014 to April 2015 (low‐bloom seasons)

NMDS analysis of the 16S rRNA gene sequence frequency data clustered on the OTU level showed a clear separation between LA and both SA and FL (Figure 4). Cluster analysis based on Jaccard's coefficient also revealed that LA was separated from SA and FL (Figure S2). Furthermore, most samples from May to November (high‐bloom seasons) were clustered and separated from that from December to April (low‐bloom seasons) in all three habitats (Figure S2). Moreover, samples collected during the same season (either high‐bloom or low‐bloom seasons) in SA and FL were much more similar to each other than among samples of the same habitat (Figure S2). ANOSIM results on the same data showed a significant difference between LA and FL (*R *=* *.911, *p *=* *.001), and LA and SA (*R *=* *.847, *p *=* *.001), whereas a small difference between SA and FL (*R *=* *.099, *p *=* *.031). Furthermore, edgeR analysis revealed that OTUs affiliated with *Cytophagia*,* Betaproteobacteria*,* Spartobacteria*, and *Actinobacteria* contributed the top five taxa to the difference between LA and FL, and OTUs affiliated with *Sphingobacteriia*,* Spartobacteria*,* Deltaproteobacteria*,* Clostridia*, and *Gammaproteobacteria* contributed the top five taxa to the difference between LA and SA (Supporting Information Table S1). When comparing the SA and FL fractions, OTUs affiliated with *Betaproteobacteria*,* Alphaproteobacteria*, and *Cytophagia* were the top five significantly different taxa (Supporting Information Table S1).

**Figure 5:**
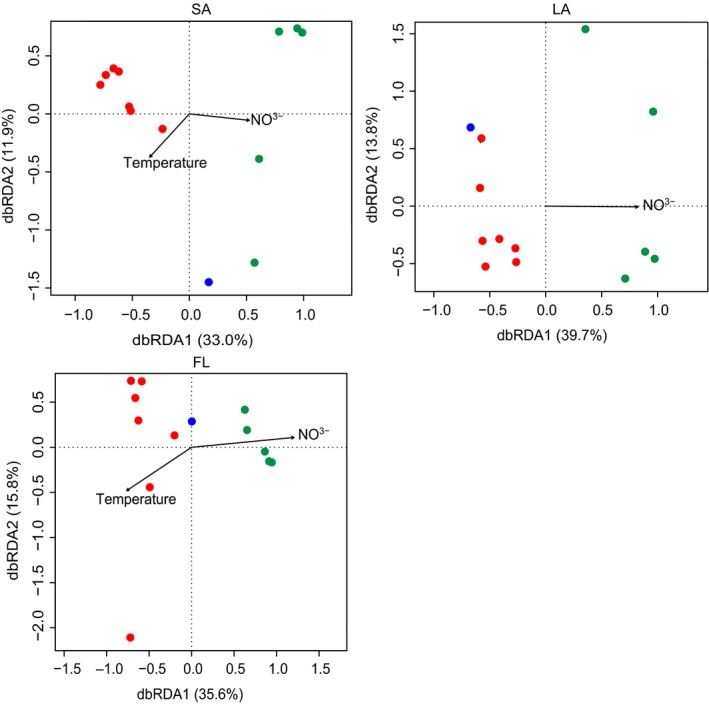
Distance‐based redundancy analysis (dbRDA) ordination plot showing the relationship between LA, SA, and FL bacterial community structures and the water environmental parameters. Numbers in parenthesis indicate the percentage of the total variance explained by the axis. Symbols in green, blue, and red indicate the samples collected from December 2014 to April 2015 (low‐bloom seasons), November 2014, and from May 2015 to October 2015, respectively

dbRDA results illustrated that NO_3_^−^ was the most significant variable (Monte Carlo test, *p *<* *.05) in the community composition of LA, accounting for 53.5% of the changes. NO_3_^−^ and temperature were the most significant variables (Monte Carlo test, *p *<* *.05) in the community composition of SA, accounting for 44.9% of the changes, whereas NO_3_^−^ and temperature were the most significant variables (Monte Carlo test, *p *<* *.05) in the community composition of FL, accounting for 51.4% of the changes (Figure 5). In addition, Mantel analysis with Bray–Curtis dissimilarity matrix revealed that cyanobacterial composition significantly correlated with bacterial composition in LA (Pearson's *r*: .798, *p *=* *.002) and FL (Pearson's *r*: .446, *p *=* *.005) but not in SA (Pearson's *r*: .084, *p *=* *0.23).

### 3.4 Dynamics of bacterial communities in the three habitats (LA, SA, and FL)

**Figure 6:**
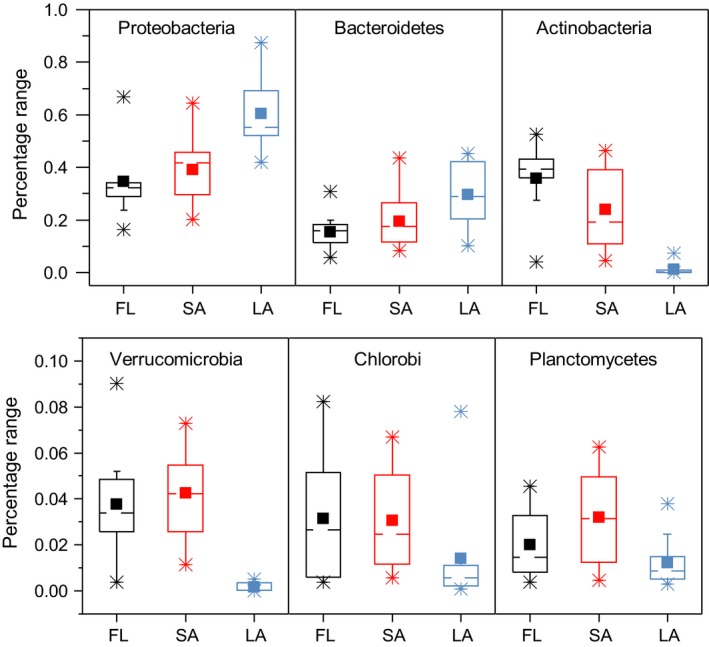
Distributions of the relative abundance (%) of the top 6 major taxa at the phylum level. The dashed line, square, and box indicate median, mean, and 25%–75% values, respectively. The diamond indicates outliers, and the whisker indicates the maximum and minimum values

In LA, bacterial communities were predominantly composed of members of the phylum *Proteobacteria* (42.0%–87.4%), followed by *Bacteroidetes* (10.1%–45.3%). *Proteobacteria* were predominant from March to August when the blooms increased, whereas the codominance of *Proteobacteria* and *Bacteroidetes* was observed from September to December when the blooms reduced (Figure S3). In SA, the bacterial communities were generally characterized by codominance of *Proteobacteria*,* Bacteriodetes*, and *Actinobacteria*, which accounted for 16.4–66.8%, 5.7–30.8%, and 4.1–52.5%, respectively. *Proteobacteria* dominated from November to April during low‐bloom seasons, as well as in September 2014 and July 2015, whereas *Actinobacteria* dominated during the other months. In FL, *Proteobacteria*,* Bacteriodetes*, and *Actinobacteria* were also the dominant phyla and occupied 22.2–64.4%, 8.2–43.6%, and 4.4–45.8%, respectively (Figure S3). The bacterial community in FL was dominated by *Actinobacteria* from July 2014 to May 2015 but shifted to the dominance of *Proteobacteria* from June to July 2015 when cyanobacterial blooms were intense. The relative abundances of *Proteobacteria* and *Bacteroidetes* were significantly higher in LA than in SA and FL (using nonparametric Mann–Whitney *U* test, *p *<* *.05), whereas others such as *Actinobacteria* and *Verrucomicrobia* were significantly lower in LA than in SA and FL (using nonparametric Mann–Whitney *U* test, *p *<* *.05) (Figure 6).

**Figure 7:**
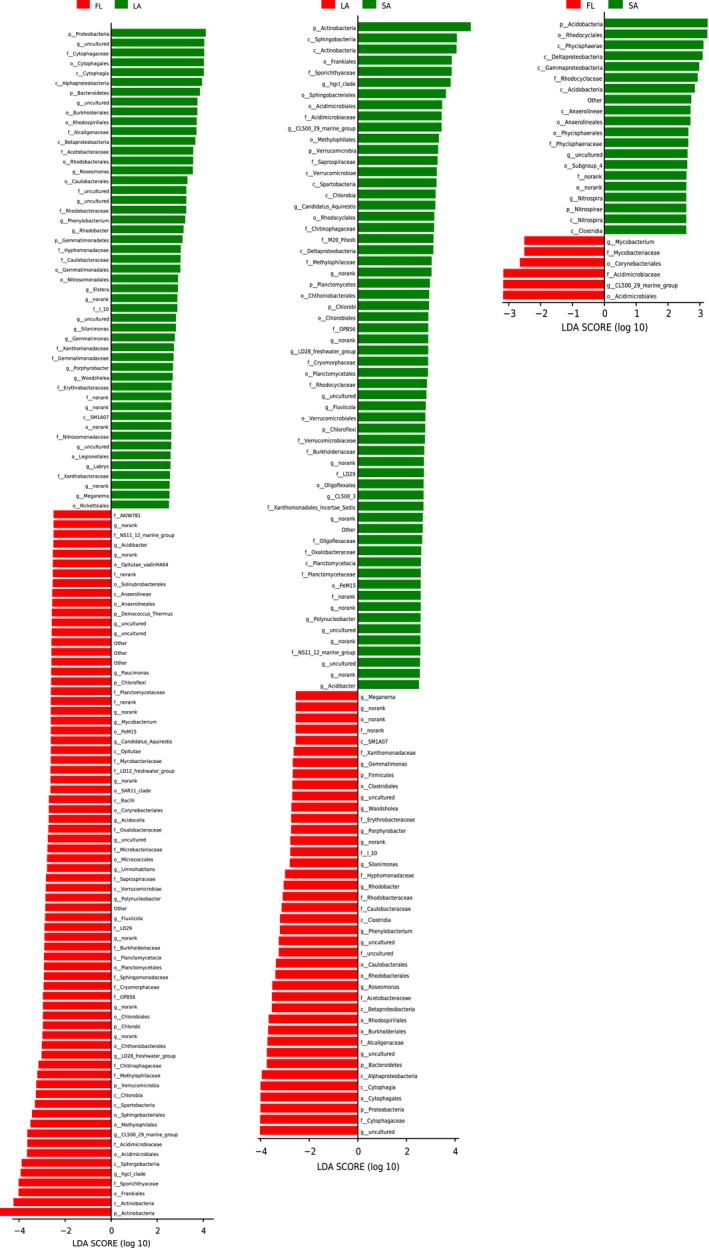
Phylum and genus are differentially represented between LA and SA (a), LA and FL (b), FL and SA (c) identified by linear discriminant analysis coupled with effect size (LEfSe) (LDA >2.5, *p* < .05). The cladogram that visualizes the output of the linear discriminant analysis coupled with effect size (LEfSe) algorithm, which identifies taxonomical differences between LA, SA, and FL community members, is shown in Figure S4

LEfSe analysis based on genus level further revealed which bacterial taxa were significantly distinct among the habitats. *Clostridia*,* Cytophagia*,* Caulobacterales*,* Xanthobacteraceae*,* Rhodobacteraceae*,* Acetobacteraceae*,* Erythrobacteraceae*,* Alcaligenaceae*,* Legionellales*, and *Nitrosomonadaceae* were enriched in LA [linear discriminant analysis (LDA) >2.5, *p* < .05], whereas *Acidobacteria*,* Saprospiraceae*,* Anaerolineae*,* Nitrospira*,* Rhodocyclaceae*,* Oligoflexaceae*, and *Verrucomicrobiaceae*, were enriched in SA (LDA >2.5, *p* < .05) (Figure 7; Figure S4). *Actinobacteria*,* Chlorobia*,* Planctomycetaceae*,* Burkholderiaceae*,* Chitinophagaceae*,* Acidimicrobiaceae*,* Mycobacteriaceae*,* Sporichthyaceae*,* Microbacteriaceae*,* Solirubrobacterales*, and *Spartobacteria* were enriched in FL (LDA >2.5, *p* < .05) (Figure 7; Figure S4). As expected, more phylotypes were found to be enriched from the comparison between LA and SA, LA and FL, than when SA and FL were compared.

## 4 DISCUSSION

### 4.1 Particle composition and dynamics of cyanobacterial composition in the three habitats (LA, SA, and FL)

Both LA and SA were examined under SEM to confirm their composition. As expected, LA was mainly composed of cyanobacterial colonies. Cyanobacteria are the predominant phytoplankton in the Meiliang Bay of Lake Taihu (Chen et al., 2003; Ma et al., 2016), and large colonies always float to the water surface. Thus, the large particles collected by exploiting buoyancy can be assumed as large cyanobacterial colonies. SA was considerably more complex and mainly composed of diatoms, single cyanobacterial cells, and other suspended particles. Thus, if one aims to focus on the bacteria attached to cyanobacterial colonies, SA must be excluded. However, few studies focused on the differentiation of the bacterial communities attached to these particles, which may coexist and be mixed.

Shifts of the dominant cyanobacterial genus from *Microcystis* in warm seasons to *Dolichospermum* in cold seasons were observed in LA. This observation may be explained by the fact that *Dolichospermum* is more favorable in lower water temperature than *Microcystis* (Robarts & Zohary, 1987). However, in SA, uncultured cyanobacteria 1 were predominant in addition to *Microcystis* in November and December, and to *Dolichospermum* in April. This finding indicated the different compositions of cyanobacterial genera in LA and SA during the most investigated months. Phytoplankton composition can shape bacterial communities (Niu et al., 2011), and high velocity of large colonies enables them to rise readily onto the surface, whereas small particles are mainly suspended in the water column (Wu & Kong, 2009; Zhu et al., 2014). Therefore, large cyanobacterial colonies and small particles would have different physiological characteristics, thereby providing different habitats for microbes.

### 4.2 Comparison of bacterial diversity in the three habitats (LA, SA, and FL)

Interestingly, all the three habitats harbored specific bacterial taxa that were not observed in the other two; this result indicates that some bacterial species have high niche specificities (Bertos‐Fortis et al., 2016). However, the number of overall OTUs that overlapped between SA and FL was higher than that between LA and FL and between LA and SA. This result indicated that the exchanges between FL and SA were relatively easy and intense, whereas the bacteria in LA were relatively isolated. Although the sampling method of sequential filtering for SA and FL and the specific colony isolation method for LA used in this study may increase the overlaps between SA and FL, significant differences between them were also observed. These results can help exclude the effect of sequential filtering to some extent and assume biological reasons for this overlap, indicating that a narrower spectrum of bacteria can thrive on large colonies. Moreover, a lower diversity of bacterial communities was observed in LA than in FL, which has lower diversity than SA. Consistent with our previous observation, the bacterial communities attached to buoyant *Microcystis* colonies had lower diversity compared with other bulk bacteria (Shi et al., 2012). These results indicated that LA was different from regular large particles, which may also harbor different communities compared with FL but had selectivity for the attached bacteria. Cyanobacteria can release antimicrobial substances surrounding algal cells (Casamatta & Wickstrom, 2000; Ostensvik, Skulberg, Underdal, & Hormazabal, 1998); thus, some bacteria may not survive in LA. These results indicated that SA composed of diverse particles harbor broader bacterial communities, whereas LA can be assumed to be enriched in selected bacterial communities to some extent.

NMDS result further suggested the distinctive microenvironment provided by LA. This result is consistent with the observation from the comparison of bacterial communities attached to size‐fractioned *Microcystis* colonies collected from August to October 2012 (Cai et al., 2014). Furthermore, LA samples dominated by *Microcytsis* were separated from the samples dominated by *Dolichospermum*, indicating that different cyanobacterial genera may also lead to separated bacterial communities, which is similar to the evidence from a previous experiment (Zhu et al., 2016). These results are consistent with our previous observation that specific bacterial communities are attached to *Microcystis* spp. (Shi, Cai, Yang, et al., 2009). Moreover, a higher similarity between SA and FL was noted during the same season (high‐bloom season or low‐bloom season) than that of the same habitat. This result indicated that sampling season also affected the distribution of these two communities. Many studies compared particle‐attached bacterial communities and free‐living bacterial communities, but controversial observations were also drawn. Some studies observed that particle‐attached bacterial communities are phylogenetically distinct from free‐living bacterial communities (Allgaier & Grossart, 2006; Zhao et al., 2017), whereas some studies observed that these two communities are similar and may exchange in freshwater mesocosms (Riemann & Winding, 2001; Tang et al., 2015, 2017; Worm, Gustavson, Garde, Borch, & Sondergaard, 2001). However, this study further concluded that particles collected during different season may be a major reason for these controversial observations. The study also evidenced the influence of cyanobacterial compositions of particles on bacterial communities. This notion is similar to the conclusion that different sizes, origins, and phytoplankton compositions may account are responsible for the discrepancies (Schmidt et al., 2016). Furthermore, particles mainly composed of LA may be more distinctive compared with general particles, which may favor distinct bacterial communities.

In addition, dbRDA revealed that NO_3_^−^ significantly correlated with the distribution pattern of bacterial community in all three habitats. The effect of NO_3_^−^ on bacterial community structure was also shown in previous studies of Lake Taihu (Tang et al., 2017), Lake Tanganyika (De Wever et al., 2005), and the mesotrophic Lake Tiefwaren (Roesel, Allgaier, & Grossart, 2012). NO_3_^−^ may directly or indirectly affect bacterial proliferation through cyanobacterial abundance and composition, which are greatly affected by nutrient concentration (Xu, Paerl, Qin, Zhu, & Gaoa, 2010). Moreover, the bacterial communities in SA and FL rely more on the temperature in the water column compared with those in LA. In addition, the samples collected during low‐bloom seasons (from December to April) formed a separate cluster from others during high‐bloom seasons (from May to November) in all the three habitats (Figure S2). A significant correlation was noted between bacterial composition and cyanobacterial composition in LA and FL but not in SA. These results indicated the seasonal dynamics of bacteria in all the three habitats and the close association of bacterial communities in LA and FL with cyanobacterial blooms. In contrast, the bacteria in SA faced a much more complex microenvironment.

### 4.3 Phylogenetic composition and dynamics of bacterial communities in three habitats (LA, SA, and FL)

The result that *Alphaproteobacteria* and *Cytophagia* dominated in LA was similar to those of studies on bacterial community attached with a diatom bloom (Riemann, Steward, & Azam, 2000). Some *Proteobacteria* and *Bacteroidetes* are well adapted to the phycosphere of phytoplankton and are specialized for successive decomposition of algal‐derived organic matter (Teeling et al., 2012). Dominance of *Proteobacteria* over an entire year indicates that these bacteria play key roles in cyanobacterial bloom formation, whereas codominance of *Proteobacteria* and *Bacteroidetes* from September to December indicates that *Bacteroidetes* also play an important role during the decline of cyanobacterial blooms. In particular, *Xanthobacteraceae*,* Rhodobacteraceae*,* Acetobacteraceae*, and *Erythrobacteraceae* are dominant in *Microcystis* cultures (Shi, Cai, Yang, et al., 2009). Many *Cytophagia* degrade macromolecules, such as proteins, chitin, pectin, agar, starch, or cellulose (Reichenbach, 2006). Most species of *Burkholderiales* utilize a variety of organic and amino acids as carbon sources (Garrity et al., 2005). Dominance of these bacteria, which vary in their ability to utilize different types of organic matters, suggested that LA may form a specific habitat, where intense bacteria algal interaction may facilitate organic matter cycling and nutrient generation, thereby benefitting algal growth.

In SA, *Actinobacteria* were predominant during most months, but *Proteobacteria* predominated from November to April when cyanobacterial blooms declined and began to form, indicating that the bacterial community in SA was also influenced by the different periods of cyanobacterial blooms. Moreover, the bacterial community in FL was predominated by *Actinobacteria* from July 2014 to May 2015 but shifted to *Proteobacteria* from June and July 2015 when cyanobacterial blooms were intense, indicating the influence of cyanobacterial blooms on bacterial community in FL. The result that *Actinobacteria* were dominant in FL was consistent with previous observations during the study of bacterial communities associated with organic aggregates in Lake Taihu (Tang et al., 2009). The result was also consistent with the results obtained from the comparison of free living and particle associated bacterial communities in the four lakes of Northeastern Germany (Allgaier & Grossart, 2006). However, our result further indicated that *Actinobacteria* are dominant in FL and in SA but not yet in LA. This result was different from some other data (Schmidt et al., 2016). Lake Taihu received a large amount of particles from surrounding terrestrial environment (Dokulil, Chen, & Cai, 2000), and these terrestrial particles may be involved in SA. Thus, the presence of *Actinobacteria*, which is well‐known from soil environments, may be one reason for its dominance in SA. These results may facilitate further recognition of the ecotypes of *Actinobacteria*.

Particularly, *Nitrospira* was reported to complete nitrification process and is a key component of nitrogen‐cycling microbial communities (Daims et al., 2015; van Kessel et al., 2015). Enrichment of these bacteria in SA indicated that the nitrification process may be involved in this microenvironment. Interestingly, proportions of *Planctomycetaceae* were significantly higher in SA than that in LA. This result was consistent with our previous observation that *Planctomycetaceae* were few in buoyant *Microcystis* colonies (Shi et al., 2012). Although close associations between *Planctomycetes* and cyanobacterial colonies were observed (Cai et al., 2013; Tang et al., 2010), free‐living *Planctomycetes* were also observed in hypoxic zone induced by *Microcystis* blooms (Li, Xing, & Wu, 2012). Actually, association of *Planctomycetes* and cyanobacterial blooms have only been observed in a lake in Sweden among the investigated 32 sites in three continents (North America, Europe and Asia) (Dziallas & Grossart, 2011), and also only in Lake Erken and Lake Limmaren among the four Swedish lakes with cyanobacterial blooms (Eiler & Bertilsson, 2004). These findings suggested that the association between *Planctomycetes* and cyanobacterial blooms is not so firm and stable.

In conclusion, this study highlights the dynamics of freshwater microbial communities in a eutrophic lake with cyanobacterial blooms during a whole‐year investigation, with regard to both the cyanobacterial and bacterial species in LA, SA, and FL. The compositions of cyanobacterial genera were different among the three habitats. The diversity of bacterial communities in LA was lower than that in FL, which was lower than that in SA. Moreover, different and more narrow bacterial communities were present in LA relative to those in SA and FL. The samples in all three habitats were collected during high‐ (May to November) and low‐bloom seasons (December to April) were separated. Furthermore, the effect of cyanobacterial composition on bacterial communities was observed in LA and FL but not in SA. This work further confirms that sampling season and particles with different characteristics may affect bacterial community composition, and that large buoyant cyanobacterial aggregates harbor specific bacterial communities. Therefore, if we take all the bacterial communities in the water column together to analyze bacterial communities associated with cyanobacterial blooms, then differences among large buoyant cyanobacterial colonies, other small particles, and free‐living bacteria may be neglected. Thus, future studies should focus on bacterial communities attached to large buoyant cyanobacterial colonies to elucidate bacterial and cyanobacterial interactions.

## CONFLICT OF INTEREST

None declared.
