# Supplementary figures and images for: Large buoyant particles dominated by cyanobacterial colonies harbor distinct bacterial communities from small suspended particles and free‐living bacteria in the water column
Source: Microbiologyopen. 2018 Mar 23;7(6):e00608. doi: 10.1002/mbo3.608 (PMC6291827; doi:10.1002/mbo3.608)

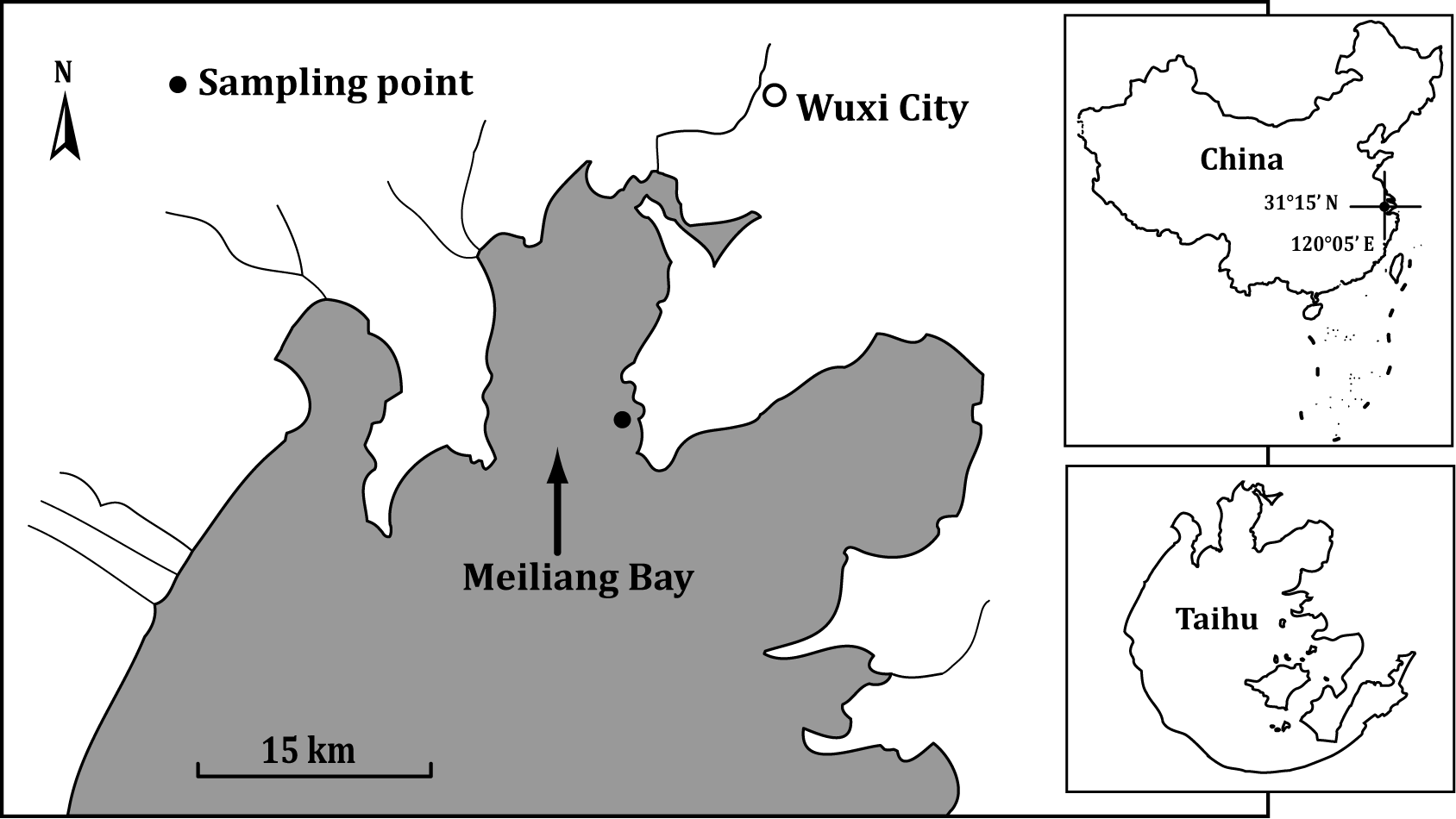

Supplement: Supplementary file 1 [file MBO3-7-e00608-s001.tif]

Jaccard's Coefficient

0.04      0.2      0.36      0.52      0.68      0.84      1

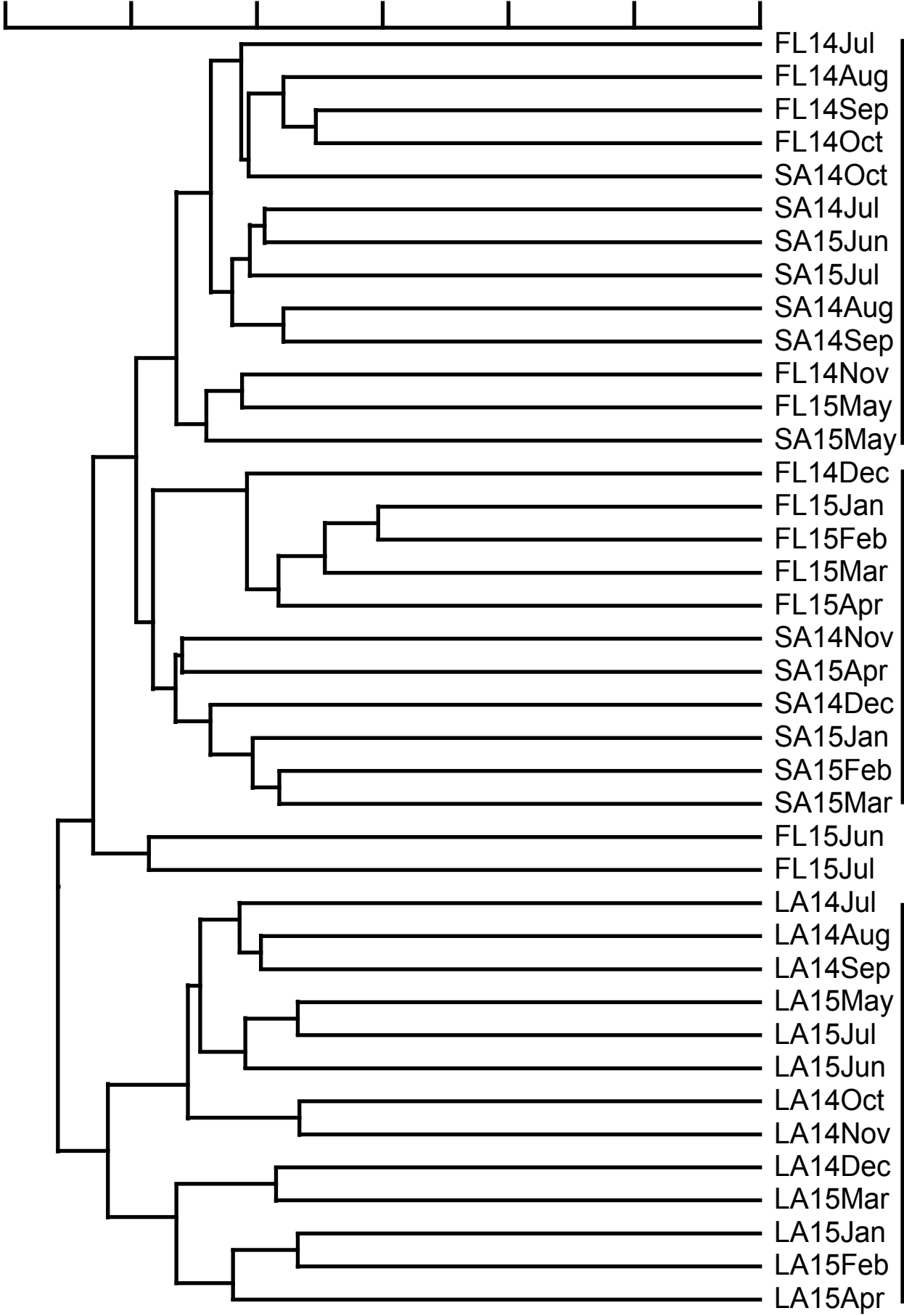

WPGMA

Supplement: Supplementary file 2 [file MBO3-7-e00608-s002.pdf]
