# Supplementary material for: Large buoyant particles dominated by cyanobacterial colonies harbor distinct bacterial communities from small suspended particles and free‐living bacteria in the water column
Source: Microbiologyopen. 2018 Mar 23;7(6):e00608. doi: 10.1002/mbo3.608 (PMC6291827; doi:10.1002/mbo3.608)
Supplement: Supplementary file 5 [file MBO3-7-e00608-s005.docx]

Supporting Figure 1 Location of the sampling station in Meiliang Bay in Lake Taihu.

Supporting Figure 2 Cluster analysis of bacterial communities in three habitats.

Supporting Figure 3 Bacterial community composition of the three habitats. Less abundant phyla (total average 0%-0.5%) and unclassified bacteria were collapsed into “others” bar.

Supporting Figure 4 The cladogram visualizes the output of the linear discriminant analysis coupled with effect size (LEfSe) algorithm, which identifies taxonomical differences between LA, SA, and FL community members. Only taxa that meet an LDA significant threshold of 2.5 are shown. Taxa with no significant differences are represented as yellow circles. The diameter of the circles is proportional to the relative abundance.

Supporting Table S1 Taxa that were significantly different between the three different habitats were detected using the Bioconductor-edgeR package. For each pair of comparison, top 5 taxa were shown.

| Var.1 | logFC | logCPM | F | PValue | FDR | Class |
| --- | --- | --- | --- | --- | --- | --- |
| FL_SA |  |  |  |  |  |  |
| OTU1539 | 6.599913 | 11.19179 | 27.62285 | 1.48E-07 | 0.00029 | Alphaproteobacteria |
| OTU2197 | 6.23767 | 10.95805 | 26.26335 | 2.99E-07 | 0.00029 | Alphaproteobacteria |
| OTU550 | -6.03596 | 10.84499 | 25.42524 | 4.61E-07 | 0.00029 | Betaproteobacteria |
| OTU916 | 5.923442 | 10.77263 | 25.01801 | 5.69E-07 | 0.00029 | Alphaproteobacteria |
| OTU3331 | -5.89388 | 10.76437 | 24.88968 | 6.08E-07 | 0.00029 | Cytophagia |
| FL_LA |  |  |  |  |  |  |
| OTU260 | -8.83518 | 14.20559 | 39.39455 | 3.48E-10 | 8.29E-07 | Cytophagia |
| OTU2184 | -8.45683 | 15.39456 | 37.45369 | 9.40E-10 | 9.09E-07 | Betaproteobacteria |
| OTU306 | 8.342929 | 13.24029 | 37.0709 | 1.14E-09 | 9.09E-07 | Spartobacteria |
| OTU168 | 7.59863 | 13.26394 | 31.88518 | 1.64E-08 | 7.83E-06 | Actinobacteria |
| OTU145 | -7.46283 | 15.51248 | 31.88249 | 1.64E-08 | 7.83E-06 | Cytophagia |
| SA_LA |  |  |  |  |  |  |
| OTU2988 | 8.232958 | 13.84302 | 35.84284 | 2.15E-09 | 5.12E-06 | Sphingobacteriia |
| OTU306 | 7.813781 | 13.24029 | 34.03077 | 5.44E-09 | 6.49E-06 | Spartobacteria |
| OTU3480 | 6.933464 | 11.93613 | 28.74479 | 8.28E-08 | 5.74E-05 | Deltaproteobacteria |
| OTU152 | -6.82018 | 11.34933 | 28.4527 | 9.63E-08 | 5.74E-05 | Clostridia |
| OTU2070 | 6.671337 | 11.94713 | 27.30659 | 1.74E-07 | 7.63E-05 | Gammaproteobacteria |
